# Predatory behavior, nesting habits, and impacts on honey bees (*Apis mellifera*) of an invasive hornet (*Vespa tropica*) on the island of Guam

**DOI:** 10.1371/journal.pone.0332986

**Published:** 2025-09-26

**Authors:** Christopher A. Rosario, Gard W. Otis, Ross H. Miller, Arianna A. Groover-Landis, Ella S. Stanley, Heather R. Mattila

**Affiliations:** 1 Guam Department of Agriculture, Biosecurity Division, Barrigada, Guam, United States of America; 2 School of Environmental Sciences, University of Guelph, Guelph, Ontario, Canada; 3 Institute of Bee Health, University of Bern, Bern, Switzerland; 4 Western Pacific Tropical Research Center, University of Guam, Mangilao, Guam, United States of America; 5 Department of Biological Sciences, Wellesley College, Wellesley, Massachusetts, United States of America; Universidade de São paulo, BRAZIL

## Abstract

Hornets (genus *Vespa*) are a conspicuous taxon of large eusocial wasps that are predators of other insects. Increasingly, hornets are gaining notoriety as damaging invaders after repeated introductions into novel habitats. Most hornets are highly effective predators, so they have the potential to greatly impact local entomofauna, including economically important pollinators. In 2016, *Vespa tropica*, a hornet with a broad natural range throughout subtropical and tropical Asia, was detected on Guam, although few details have been published since the initial alert. We provide the first comprehensive update on the status and impact of *V. tropica* hornets on Guam based on nine years of beekeeper and public reports, as well as field collections of nests, hornets, and videos of hunting behavior in managed apiaries. We show that the population of non-native *V. tropica* is established and thriving on Guam. Nests were found in a diversity of sites: below and above ground, sheltered and exposed, and in urban and greenspaces. *V. tropica* was a year-round predator in apiaries, with up to 12% of colony losses per year attributed to hornet attacks. Notably, hornets often attacked single honey bee (*Apis mellifera*) colonies in groups, similar to the hunting strategy of their close relatives, the giant hornets *Vespa mandarinia* and *Vespa soror*. Hornets killed defending workers, eventually weakening colonies and entering hives to consume bee brood. Bees mounted defenses that included alarm piping, bee ‘carpets’, and attempts to ball hornets. Hornets were active all year, but were significantly more active during the wet season (July‒December). Preliminary analysis of color forms suggested that the source of *V. tropica* on Guam is likely continental southeast Asia, although genetic analyses are required. Our study reveals that Guam is facing a fierce invader in *V. tropica*, which is placing strong predatory pressure on pre-established honey bees.

## Introduction

The accidental translocation of hornets (genus *Vespa*) out of their native ranges and into novel habitats, spurred by increases in global trade, has given rise to many invasive hornet populations around the world. Of the 22 recognized species of hornets, most of which are naturally restricted to Asia [1–3], nine species have been documented in countries outside of their native range and five species have proven over time to have established thriving invasions as non-natives, with several species doing so in multiple parts of the world [4–6]. The more frequent such hornet invasions become, the more attention they garner because of the ecological damage and economic costs they are predicted to cause [7–11].

Several aspects of the biology of hornets make them strong candidates to become impactful invaders in new ecosystems [4,8]. First, all hornets have an annual cycle that ends with the production of inseminated gynes that are capable of founding new nests on their own [12]. These young gynes diapause in a sheltered hibernaculum after they mate, so solitary, inactive females can easily and inadvertently evade detection as stowaways in shipped goods [4,8]. Secondly, most hornets are generalist or semi-specialist insect predators [12,13], and evidence shows that invasive hornets can pivot to hunting novel prey species in new ecosystems [14–19]. Colonies consume substantial prey biomass to support colony growth, especially toward the end of their annual cycle when colonies are most populous and future reproductive are being reared [13]. Moreover, many hornet species target other social insects as prey [13,20], which suggests they may have an outsized impact on economically important pollinators [21]. *Vespa velutina* exemplifies a worst-case scenario for a hornet invasion [11,22–24]. Described as a semi-specialist predator, a single *V. velutina* colony consumes more than 11 kg of insect biomass, the majority of which includes honey bees and other social wasps [14,16,18,19]. *V. velutina* has already established invasive populations in Japan, South Korea, and western Europe [4], and recently colonized the United States [5]. A single founding event in France has fueled an expanding invasion across Europe and the United Kingdom that is causing widespread ecosystem effects [25–29].

The University of Guam released a brief bulletin that reported the detection in 2016 of *Vespa tropica* hornets and nests from three locations on Guam, a United States island territory in the Mariana Islands Archipelago [30]. This was the first documented case of the genus *Vespa* in Micronesia [30]. No other information has been published about the biology of non-native *V. tropica* on Guam since this initial report. A recent survey of the health of wild and managed honey bees on Guam briefly described attacks on managed honey bee colonies by *V. tropica* hornets as an ongoing and widespread threat to beekeeping [31]. Here, we provide the first description, in several dimensions, of the nesting and predatory habits of non-native *V. tropica* hornets nine years after they were first discovered on Guam. We preface this information with a summary of the known biology of this large hornet in its native range, with consideration for factors that could amplify its threat as an invasive.

### Biology of *Vespa tropica* in its native range

Information about the biology of *V. tropica* has been published over decades, but it has been obfuscated by confusion about species identity. Notably, extensive descriptions were written by early influential hornet experts, and the biology of “*Vespa tropica pulchra*” was especially well documented in Japan [12,13,20,32]. However, this northern form was later recognized as a distinct species, *Vespa ducalis*, a closely related species in the ‘tropica’ group that produces substantially smaller colonies than *V. tropica* [33,34]. Although *V. tropica* is an impressively widespread species, it does not occur in Japan. In the tropics, early authors also incorrectly collapsed the separate species *Vespa affinis* and *V. tropica* into the no-longer-recognized *Vespa cincta*, forcing later authors to sleuth their biological differences [20,35]. Thus, details of *V. tropica* biology are somewhat scattershot across the literature as they have been reported piecemeal across its broad range. For this reason, we start our update on the invasion of Guam by *V. tropica* hornets with a brief synthesis about what is currently understood about the species within its native range.

*V. tropica* is naturally distributed across a broad swath of tropical and subtropical Asia, from Afghanistan at its western reach to New Guinea in the east, including the Indian subcontinent, continental southeast Asia, southern China, most of Indonesia, and the Philippines [1,20,33,36]. The many color forms of *V. tropica* across its large range have created taxonomic confusion over time as authors categorized local populations as varieties, races, subspecies, and sometimes species based on color alone [20,35,37–40]. Archer [33] separated some color forms from *V. tropica*, attributing them instead to *Vespa ducalis*, and began to move away from the use of subspecies, a taxonomic approach that has been affirmed by subsequent keys based on morphology [1,2,41]. A genetic survey of *V. tropica* has never been conducted. Thus, color forms, as most recently defined by Archer [33], provide the only current means of distinguishing individuals from different regions.

While *V. tropica*’s preferred habitat has not been well characterized, it appears to flourish in low-elevation zones with warm, humid climates. It has been described as a common inhabitant of tropical lowland and hilly districts, no more than 800 m above sea level in Java [20], and in grasslands, forests, and disturbed agricultural spaces in Malaysia, Sumatra, Borneo, Singapore, and islands eastward to New Guinea [20,35,36,42,43]. It is also been described as commonplace in the lowland terai plains and thick forests of India [44,45]. The ability of *V. tropica* colonies and diapausing gynes to tolerate temperate conditions within its native range is not clear, but records suggest it is restricted to subtropical and tropical zones [46].

*V. tropica* has several nesting behaviors that may increase its potential to be a damaging invasive species, although not all traits are well documented. Importantly, it is clear that *V. tropica* has flexible nesting habits. While it is most commonly found in enclosed spaces, its nests have been reported in both subterranean and above-ground locations (in natural hollows or sheltered human structures) and, occasionally, aerial or exposed nests have been found [35,36,38,42,43,45,47–50]. In Singapore, it has also been reported as being more commonly associated with human-dominated landscapes relative to co-occurring *Vespa* species [48], although it was described as much less prone to human affiliation in Singapore and Sumatra by others [36,42]. While the growth of *V. tropica* colonies has not been closely monitored, it apparently adheres to the annual cycle that characterizes all *Vespa* species, in which new gynes found nests and rear the first generation of workers that thereafter take over colony tasks [12,51]. This cycle culminates in the production of reproductives of both sexes, with only inseminated gynes surviving to establish new colonies at the start of the next cycle. For tropical species that have been studied, there is typically a lack of seasonality to the phases of colony growth: *Vespa analis*, *V. affinis*, and *V. velutina* have been found at various stages of development throughout the year in Java and Sumatra [20,42,51]. By extension, inseminated *V. tropica* gynes in tropical regions may also be produced at any time during the year. While individual *Vespa* colonies in the subtropics and tropics tend to complete their annual cycle within a year, their extended period of growth allows colonies to become larger relative to temperate conspecifics or species limited to temperate zones [12,42,51,52]. It is not known how closely *V. tropica* follows these tropical trends. Finally, many tropical *Vespa* colonies are polygynous and pleometrotic, with multiple queens present at nest founding, in contrast to the monogynous (and haplometrotic) state that is well known for temperate vespines [42,43,51,53,54]. Matsuura [42] reported 2–6 queens in the *V. tropica* nests he examined in Sumatra and Banu and Huda [49] closely observed a single *V. tropica* queen that was joined by two other queens while founding a nest in Malaysia. Polygyny is believed to confer many benefits to social wasps, including improved nest homeostasis and higher survivorship at the founding stage, as well as faster colony growth [42,49,54–58], all of which could increase *V. tropica*’s potential threat to ecosystems as an invasive species.

While there is limited information about the size and reproductive capacity of *V. tropica* colonies, available reports describe relatively large colonies at maturity. Matsuura [42] censused two mature colonies in Sumatra, both with four combs. One nest had 2,819 cells, 845 workers and no reproductive adults present yet, and the other had 5,667 cells and 1,018 adults present, including 245 gynes. Kojima [38] described a three-comb *V. tropica* nest of unknown maturity in the Philippines that had 2,580 cells. Matsuura and Yamane [12] estimated that huge *V. tropica* colonies in Sumatra likely produced 500–1,000 gynes per colony, and they marveled at the biomass that would be necessary to sustain such large hornet colonies. Information is scant, but available data suggest potentially high reproductive capacity per colony, if there is sufficient prey to support growth.

**Fig 1:**
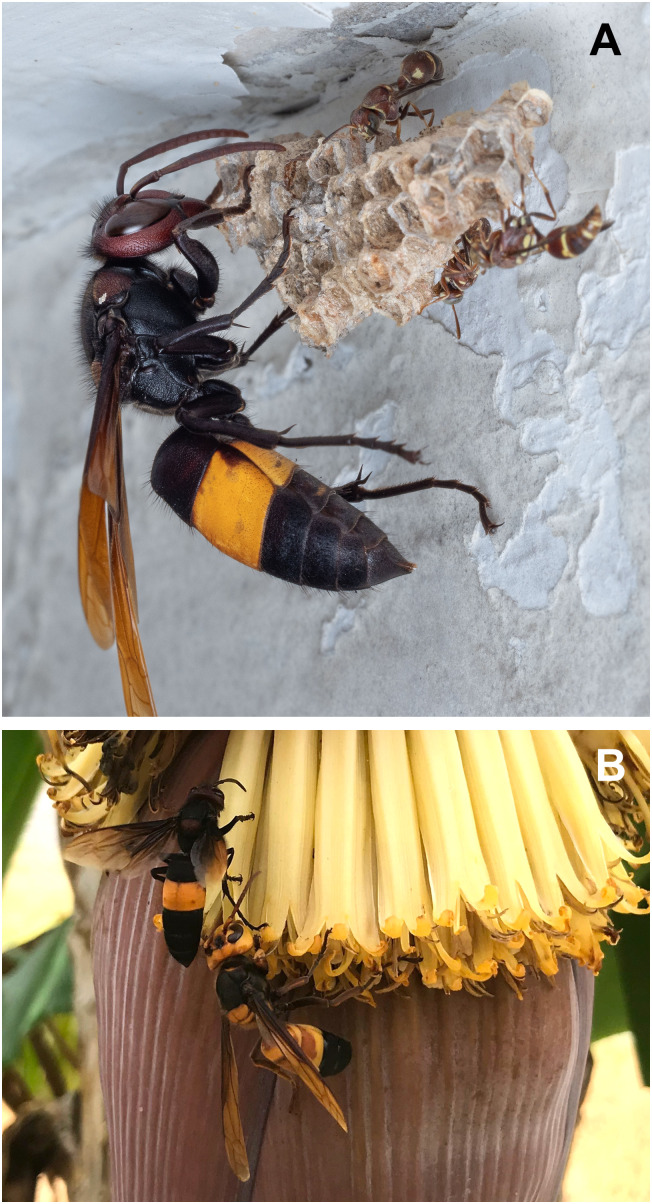
*Vespa tropica* workers foraging. (A) *V. tropica* worker attacking a small colony of a eusocial wasps (*Ropalidia* sp.) near Bengaluru, India (photo courtesy of V. Kumar). (B) *V. tropica* worker, left, compared to a giant hornet *Vespa soror* worker, right, on banana flowers in Hong Kong (photo courtesy of J. Yang).

Most *Vespa* species are described as generalist predators of insects [51], but *V. tropica* hornets are considered one of the more specialized predators in the genus. They are thought to predominantly hunt other social wasps, having been observed preying upon *Parischnogaster*, *Liostenogaster*, *Stenogaster*, *Polistes*, *Parapolybia*, and *Ropalidia* nests by multiple authors [20,42,59,60]. All authors describe attacks as a lone, large hornet opening cells to consume prey larvae, while smaller adult victims usually move off the nest to avoid interacting with the attacker (Fig 1A; see S1 Supporting Information for all photo information). Matsuura [42] specifically mentioned that he did not see *V. tropica* hornets attacking other abundant *Vespa* species, honey bees, or stingless bees in Sumatra. However, other accounts detail unrelenting attacks on honey bee colonies. In India, *V. tropica* hornets repeatedly attack managed apiaries of *Apis mellifera*, with hornets catching adult bees in front of hives and carrying them away [45,61–63]. There are also uncommon reports of group attacks on honey bee colonies, similar to those of giant hornets (Fig 1B, [13,64,65]). Specifically, in Thailand, Seeley et al. [66] described an attack on an *Apis florea* nest in which three *V. tropica* hornets killed defending workers, forcing the bees to abscond after two days of attack. Burgett and Akratanakul [67] detailed an attack on an *A. mellifera* colony that escalated to the involvement of 25–35 hornets over four days. Both of these latter attacks ended with occupation of the bee nest by the hornets, which subsequently carried away brood [66,67]. Other accounts also provide brief details suggesting that group attacks by *V. tropica* on *Apis* colonies occur [36,62,68]. Other than these accounts, no information is available about the diversity of *V. tropica*’s diet. Close study of invasive *V. velutina* revealed that it is a cosmopolitan predator with a strong predilection for honey bees and social wasps [14–16,18,19]. The extent of *V. tropica*’s predatory palate remains unclear, but a focus on predation of bees and social wasps is expected, the availability of which will impact its role as a predator in a non-native ecosystem [69].

With these details about the biology of *V. tropica* in its native range in mind, we elaborate on its nesting habits, predatory behavior, and impact on managed honey bees over the last nine years as an invasive on Guam. We also compare the color forms of *V. tropica* on Guam to the color variation documented throughout its native range as a preliminary step in determining the geographic source of Guam invaders as a genetic database is assembled. Our findings highlight intriguing predator-prey interactions between invasive hornets and introduced honey bees, which are affecting how beekeepers manage hives on Guam.

## Materials and methods

### Nesting habits of *V. tropica* on Guam

After the discovery of the first *V. tropica* hornets in July 2016, Guam residents were encouraged to report nests to representatives from the Guam Beekeepers Association, the University of Guam, and the Department of Agriculture Biosecurity Division (via the Invasive Species ‘Pest Hotline’, a program that is widely known on Guam). One of us (CAR) collated reports from 2016 onward and either confirmed nest details in person (majority of reports) or by phone with the reporting individual when a site visit was not possible. For each nest, the date of detection, habitat type (greenspace versus residential/urban), and details of the nesting site (above or below ground, exposed or sheltered, human-made or natural cavity, as applicable) were recorded. If the nest was associated with vegetation, the plant species was noted, if known. If above ground, the height of the center of the nest or cavity opening was estimated in foot-long increments (estimated visually or using a tape measure if the situation allowed), which was subsequently converted to meters.

After detection, nests were destroyed at night either by one of us (CAR) or a professional pest control company, if circumstances permitted. In several instances, intact nest materials were recovered and cell counts were estimated (by directly counting the number of cells in the first nest and then extrapolating based on total comb area for subsequent nests). On one occasion, the number of adult hornets was also counted. In most cases, nest materials were ignited with a propane torch and therefore could not be salvaged.

We compared categorical nest details based on reports made from 2016 to 2024 via chi-square tests, including the number of nests found during the dry season (January to June) versus the wet season (July to December), seasonality that characterizes Guam’s marine tropical climate [70]. All statistical tests were performed using SAS (v. 9.3, SAS Institute).

### Predatory behavior of *V. tropica* on Guam

Similar to the reporting of *V. tropica* nests, Guam residents were encouraged to report observations of active adult hornets and losses of managed honey bee colonies attributed to hornet attacks. Colony losses, as assessed by beekeepers, were individually logged by the state apiary inspector (CAR) in apiaries from 2016 onward to the end of 2024. Losses were summed per year and compared to the total number of managed hives on Guam, which was tracked annually by the Guam Beekeepers Association. We also collated observations beekeepers made about colony conditions when they discovered a loss, such as the presence of living or dead hornets and bees inside or outside of a hive. In addition to colony losses, beekeepers also reported sightings of hornet adults in their apiaries from 2017 onward. Their reports included details such as the date of observation, whether hornets were attacking colonies upon detection and, if so, how many hives were targeted and whether a single hornet or multiple hornets were involved (some reports included specific numbers of hornets). Additionally, members of the general public called the Guam Department of Agriculture’s Pest Hotline from its initiation in 2020 onward to report sightings of invasive organisms, which included *V. tropica*. We examined the total number of these reports made per month to determine whether there was seasonality in hornet activity (compared via chi-square test). Because the names of reporting individuals were also recorded, we confirmed that hornet sightings were not duplicated between reporting agencies before reports were anonymized for this analysis. Although collection of hornet reports is ongoing, we examined full-year data only, so all counts ended in December 2024.

To confirm beekeeper reports of the predatory behavior of *V. tropica* hornets, we assembled a robust set of video recordings and pictures (> 250 files) made from 2017 onward that documented hornet-bee interactions at managed hives during hornet attacks on Guam (n = 24 attacks). Some of these videos and images were shared by beekeepers and others were recorded by us (CAR and GWO). Of the multiple-hornet attacks that were videorecorded (n = 7 attacks), the longest recorded attack lasted for eight consecutive days (until the beekeeper killed the hornets to save the colony). For this attack, we recorded the entrance with two digital video cameras (Sony Handycam, model HDRCX405) that were on tripods and positioned so that they had front and side views of the hive’s entrance and its surroundings, as well as close-up videos that were taken with a cell phone (Apple, iPhone 13 Pro Max). For videos in this series, the available footage (162 min of attack) was examined to determine the number of hornets present, the number of attempts made by hornets to grab bees, the number of grabs that were successful, and the number of bees that were carried away (versus dropped in the vicinity of the hive) per minute. Across all videos, we also determined frequency of trophallaxis between attacking hornets when they were landed on hives and the number of attempts by honey bees to ball hornets. Several cell phone videos were close enough to hive entrances that audible pipes could be heard and piping honey bee workers observed. Audio from these video recordings was analyzed using Raven Pro version 1.5 [71] to determine the duration and frequency structure of vibroacoustic signals, allowing comparison with signals produced by *Apis* in other contexts.

### Potential origin of *V. tropica* on Guam

Hornets (*Vespa* spp.) exist in numerous regional color forms across their ranges that, coupled with extensive Müllerian mimicry between species, has resulted in considerable taxonomic confusion over the past century [3,41]. Van der Vecht [20,37], after reviewing the color forms identified by Bequaert [35], recognized 12 forms that he referred to as subspecies of *V. tropica*. Several of these subspecies were subsequently synonymized or recognized as other species, resulting in eight *V. tropica* color forms (formerly subspecies) recognized by Archer [33] and other authors thereafter [1,3,41]. Body-color variations tend to grade into one another for continental *Vespa* species [3], but some of these color forms, particularly those restricted to islands, are unique and may offer preliminary insight into the geographic origin of invasive *V. tropica* on Guam, in the absence of a genetic database for *V. tropica* in its native and invasive ranges. An effort to collect and genetically analyze specimens is ongoing.

Using the geographic distributions described by the above authors for these *V. tropica* color forms, we searched iNaturalist.org [46] for high-quality color photographs of seven of the color forms: Indian-Chinese (*haematodes*), Malayan (*leefmansi*), north Philippine Islands (*deusta*), south Philippine and Palawan Islands (*anthracina*), Sulawesi to New Britain (*trimeres*), Java and Bali (*tropica*), and Andaman Islands (*eulemoides*), excluding Buru (*unicolor*, for which there were no iNaturalist records), as described by Archer [33]. We categorized the color of the head, scutellum, pronotum, and first three gastral tergites in randomly selected iNaturalist images in which these features were clear. We examined a total of 239 iNaturalist images from *V. tropica*’s native range and 70 images from Guam. Additionally, we examined 69 worker specimens collected on Guam and field videos in which the body coloration of 20 hornets was clearly visible. If a body segment was a mix of colors, then we recorded the dominant color as red, black, or orange, following Archer [33]. We excluded data for individuals for which we could not see all three scored characters on either the head+thorax or the gaster. Color profiles for these body parts were compared between Guam hornets and those within the geographic distributions of the seven color forms listed above. Publicly deposited latitude and longitude coordinates for 2,112 research-grade observations of *V. tropica* [46] were mapped using QGIS (version 3.40.9 ‘Bratislava’) to illustrate its native and invasive ranges.

### Ethics statement

No protected species were harmed during the course of this field study. *V. tropica* is a damaging, invasive species on Guam that beekeepers often kill as a means of protecting managed honey bee colonies, so permits were not required to sample nests or obtain hornet specimens. Nests were removed once detected if circumstances permitted it to protect public safety. All activities with beekeepers were undertaken with their permission to access private property. Guam residents who observed nests or adult hornets gave permission to agency officials to go on their land to confirm reports (CAR and RHM are local government officials who carry agency credentials: CAR is the Head of the Biosecurity Office of the Department of Agriculture and the State Bee Inspector; RHM is an inspector for the United States Department of Agriculture Animal and Plant Health Inspection Service). All reports made to local officials or agencies were anonymized for this study. Anonymized reports and all other study data are available in S2 Dataset.

## Results

### Nesting habits of *V. tropica* on Guam

**Fig 2:**
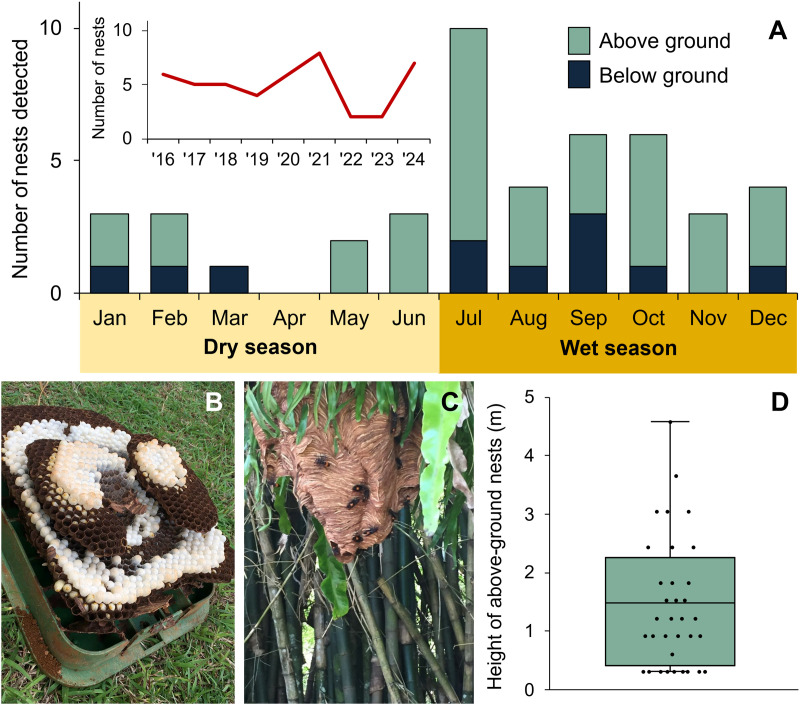
Detections of *V. tropica* nests on Guam. (A) Number of reports made by the public of *V. tropica* nests, by month from 2016 to 2024 (inset: number of reports made by year). (B) A below-ground nest constructed in the buried box of an irrigation system. (C) An above-ground, exposed nest in a bamboo patch. (D) Heights of 34 above-ground nests.

From 2016 through 2024, 45 *V. tropica* nests were reported by Guam residents. The majority of nests were discovered during the wet season (χ^2^ = 9.8, df = 1, **p* *= 0.0018), and particularly during the transition to the wet season (the month of July), rather than during the dry season (Fig 2A). Nests were found every year since *V. tropica* was first detected on Guam in 2016 (Fig 2A, inset).

Nests were discovered in a variety of locations, although most nests were either in a cavity or sheltered space compared to exposed locations (41 versus 4 nests; χ^2^ = 30.4, df = 1, **p* *= 0.0001). Also, it was more common to find nests above ground rather than below ground (34 versus 11 nests; Fig 2A; χ^2^ = 11.8, df = 1, **p* *= 0.0006). Almost all of the 11 below-ground nests were found in naturally occurring cavities: 5 nests were in subterranean cavities associated with rotting trees or cycads (e.g., *Cocos nucifera*, *Cycas micronesica*, *Casuarina equisetifolia*), 4 nests were in rock cavities (including in a quarried cliff face, which we categorized as a ‘below’ ground, vertical substrate), 1 nest was in an excavated cavity in the dirt, and 1 nest was inside the buried box of an irrigation system (Fig 2B). Of the 34 above-ground nests, 10 nests were found in hollows of several different tree and cycad species (e.g., *Persea americana*, *C. micronesica*, *C. equisetifolia*, and *Vitex parviflora*) and 20 nests were in human-made cavities (14 nests were enclosed in walls, ducts, poles, containers, and furniture) or protected in low-use spaces (6 nests were in garages or rooms of abandoned homes). The remaining 4 above-ground nests were exposed: 1 nest was hanging in a dense patch of bamboo (*Bambusa vulgaris*, Fig 2C) and the other 3 nests were attached to trees (*Hibiscus tiliaceus* and *Mangifera indica*) or a tree fern (*Cibotium menziesii*). The average height of above-ground nests was 1.4 m (Fig 2D), which may be a low estimate because of a reliance on public detection. We note that 5 of the 34 above-ground nests were in tree cavities with entrances not far from the ground (0.3 m; Fig 2D), so their nests may have extended below ground level.

Nests were more often detected in residential or urban spaces associated with private and public properties rather than in greenspaces, which included forests, grasslands, farms, or golf courses (33 versus 12 nests; χ^2^ = 9.8, df = 1, **p* *= 0.0017). Like nest heights, these counts may reflect a bias inherent to public reporting.

On four occasions, nest materials of above-ground nests were recovered completely and nest sizes were estimated. One nest collected in May 2023 had 754 adults in it and approximately 800 cells. Another nest extracted in June 2022 had 650 cells. Two nests collected in July 2021 had approximately 1,200 and 1,350 cells each.

### Predatory behavior of *V. tropica* on Guam

**Fig 3:**
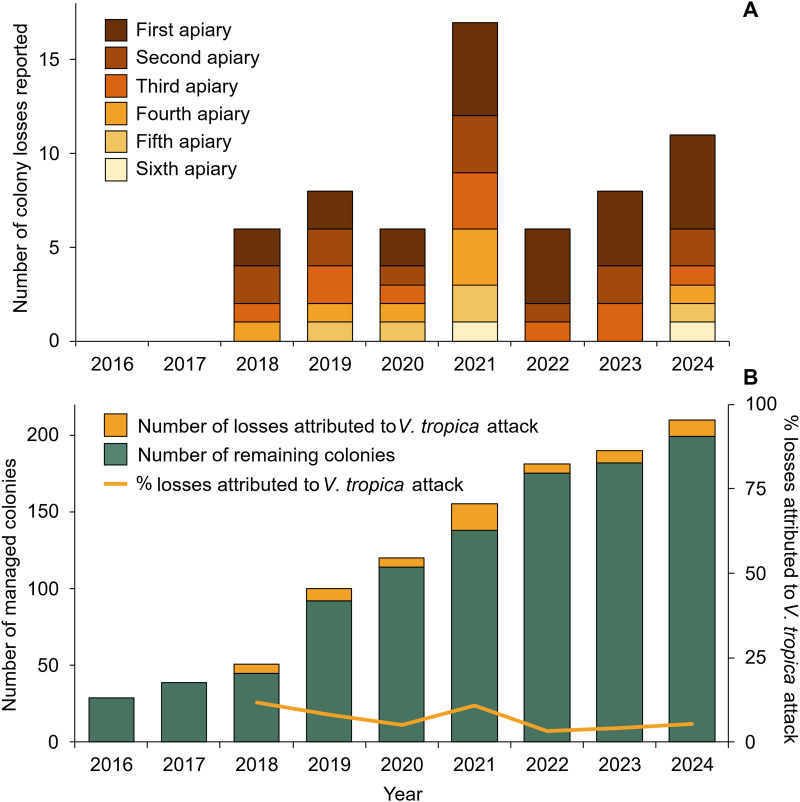
Reported losses of managed *Apis mellifera* colonies on Guam due to *Vespa tropica* attack. (A) Per reporting apiary, the number of managed honey bee colonies that were considered an economic loss due to attack by *V. tropica*, from the first detection of *V. tropica* in 2016 until the end of 2024. (B) The number of colonies reported lost due to *V. tropica* attack out of the total number of managed hives per year (left y-axis), which yielded the percentage of managed colonies for which loss was attributed to *V. tropica* attack (right y-axis).

**Fig 4:**
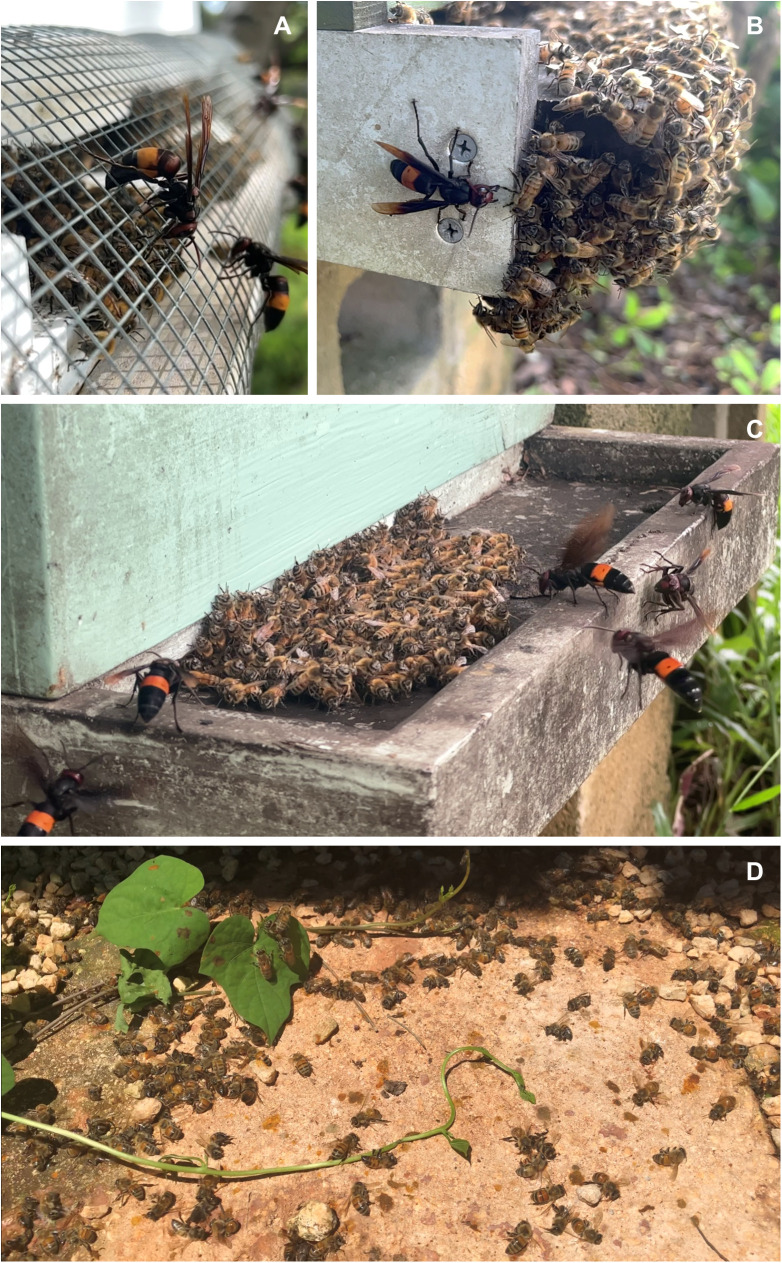
Predatory behavior of *Vespa tropica* workers. (A) Many beekeepers screen hive entrances to prevent hornet entry. (B) A *V. tropica* worker lunging at *A. mellifera* workers on the periphery of a bee carpet. (C) Multiple hornets attacking honey bees in a bee carpet. (D) Dead bees discarded by hunting hornets in front of a hive.

Beekeepers began to report losses of managed *A. mellifera* colonies that they attributed to attacks by *V. tropica* hornets in 2018, two years after hornets were first detected on Guam and one year after they were first observed hunting at hives (Fig 3). From 2018 onward, beekeepers have attributed the loss of a total of 62 managed colonies to *V. tropica* attacks, with 6–17 colony losses reported in ≤6 apiaries per year (Fig 3A). Reported colony losses due to *V. tropica* predation have accounted for as much as 12% of all managed hives annually (e.g., 2021), although this percentage declined as the number of managed hives increased (Fig 3B) and beekeeper interventions to protect colonies improved. An extensive education campaign by the Guam Beekeepers Association and the University of Guam has enhanced beekeeper vigilance and diversified hive defenses, including bait trapping with honey comb, screening and trapping at hive entrances (Fig 4A), and killing hornets as they hunt in apiaries, all of which has made beekeeping more time consuming on Guam.

**Fig 5:**
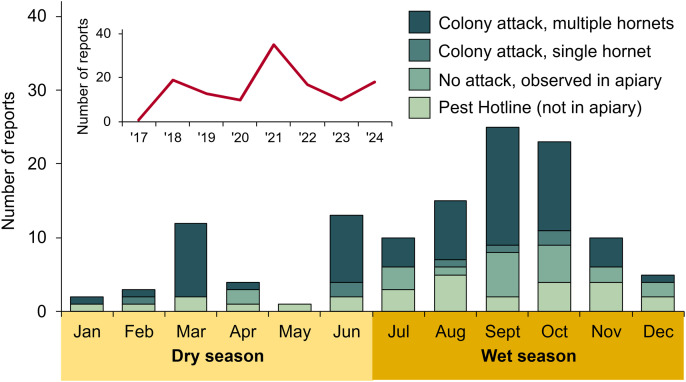
Seasonal pattern of *V. tropica* adult activity. Number of reports per month and year (inset) were summed across reporting constituencies. Beekeepers reported hornet sightings in apiaries to the Guam Beekeepers Association; their reports covered 2017–2024 and ranged from single and multiple-hornet attacks on colonies to no attack (e.g., flying around the apiary). Outside of apiaries, the general public reported adult hornet sightings to the Pest Hotline; those reports covered 2020–2024.

Collated reports from beekeepers and the general public of *V. tropica* hornet sightings in and outside of apiaries, respectively, showed a seasonal pattern of activity for hornet adults that mirrored the discovery of *V. tropica* nests (Figs 2A and 5). Adult workers were observed and reported more often by Guam residents during the wet season compared to the dry season (χ^2^ = 22.8, df = 1, **p* *= 0.0001), with 72% of reports made during the latter half of the year. Attacks in apiaries were reported every year from 2017 onward (Fig 5).

We examined observations reported by beekeepers, either when assessing a colony loss (58 reports) or when *V. tropica* hornets were actively attacking bee colonies (74 reports), the details of which helped to describe the role of *V. tropica* attacks in reported colony losses. Collectively, these observations paint a picture of *V. tropica* predation on Guam that is aligned with the predatory behavior of hornets that attack in groups (which we confirmed with field videos; see below). The majority, or 91%, of active attacks that were reported by beekeepers included the presence of multiple *V. tropica* hornets, and almost half of these attacks, or 43%, were directed at a single colony in an apiary. The number of hornets observed at a single hive ranged from 2–25 individuals, based on beekeeper reports that recorded hornet number or field videos (mean 7.3 hornets, n = 10 multiple-hornet attacks). Across all 132 reports, beekeepers often described the formation of a tightly packed ‘carpet’ of bees at hive entrances (37 reports) from which hornets grabbed bees (18 reports) (Figs 4B and 4C). Hornets were observed going in and out of hives (22 reports), leaving dead adult bees in front of hives (13 reports; Fig 4D), and chewing their way into hives (11 reports). Colony losses ranged from a colony being so weakened that it was no longer viable to the disappearance and presumed absconding of adult bees (29 reports of presumed absconding). Beekeepers reported foraging paralysis associated with colony loss (4 reports). Beekeepers, upon assessing a colony loss, found hornets inside hives eating bee brood on 22 occasions. Multiple-hornet attacks occurred more than twice as often during the wet season than the dry season (Fig 5; 45 reports versus 22 reports; χ^2^ = 7.9, df = 1, **p* *= 0.005).

We confirmed reports of multiple-hornet attacks with video recordings that ranged in duration from 35 s to 162 min of footage per attack. They showed attacking hornets hovering in the air in the vicinity of hive entrances, landing at the edge of assembled bees, and lunging with open mandibles at individual workers (Figs 4B and 4C, S3 Video). Hornets were not observed catching bees in flight. When a hornet captured a bee, it either carried it out of the field of view or killed and dropped it without leaving the vicinity of the hive, sometimes repeatedly within the same minute (S4 Video). Video from the 8-day, multiple-hornet attack showed 1–8 hornets (mean 3.7 hornets/min) present simultaneously at the same targeted hive. During 162 minutes of footage, hornet attackers made 1,143 attempts to grab worker bees, 199 (17.4%) of which were successful. Only 35 bees were carried out of the field of view of the camera; the rest were immediately dropped in front of the hive. Over a 19-minute period, when this attack was at its most intense, up to 8 hornets made 516 attempts to grab bees (mean 27.2 bees/min), although only 131 bees (25.4%) were caught. Trophallaxis between co-attackers was observed 38 times in videorecordings, an affiliative behavior that suggested cooperation and not competition among hunting hornets (S5 Video).

**Fig 6:**
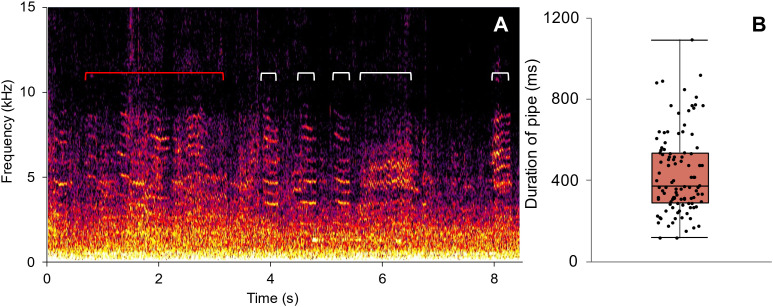
Workers in *A. mellifera* colonies piped repeatedly during *V. tropica* hornet attacks. (A) Spectrogram of *A. mellifera* workers piping when *V. tropica* attackers were hunting at a hive entrance; audio is from a cell phone recording made ~0.5 m from a hive entrance. White brackets indicate pipes that were clear enough to determine pipe duration; the red bracket indicates a series of many overlapping pipes. (B) Duration of 108 isolated pipes recorded at five hive entrances during hornet attacks.

When one or more hornets was present, honey bee workers tended to assemble outside the hive, often forming layers of bees that extended up the front wall of the hive and over the landing board in front of the entrance (Fig 4C). In several videos, the camera was close enough to the bees that it recorded audible pipes when one or more hornets were present (S6 Video). When piping, a worker pressed her thorax on a nestmate below her or on the substrate and vibrated her wings very briefly (S7 Video, S8 Video). Piping workers moved through the clustered bees outside their hive entrances and often approached the front line of the attack, signaling repeatedly as they walked, with several signals audible per second in the recording (Fig 6A). Analysis of pipes showed they had a mean duration of 432 ± 199 ms (n = 108 pipes examined from five different field videos; Fig 6B) and had a harmonic structure. Because of ambient noise due to recording conditions, the fundamental frequency of pipes could not be determined. We also observed bees attempting to ball hornets three times; each balled hornet escaped (S9 Video). Although we did not videorecord a bee ball that successfully killed an attacking hornet, beekeepers, including one of us (CAR), have discovered dead hornets inside hives after attacks.

We also observed *V. tropica* engaging in solitary-hornet attacks on small nests of eusocial wasp prey on Guam, which included *Polistes stigma*, *Polistes olivaceus*, and *Ropalidia marginata* (S10 Video).

### Potential origin of *Vespa tropica* on Guam

**Fig 7:**
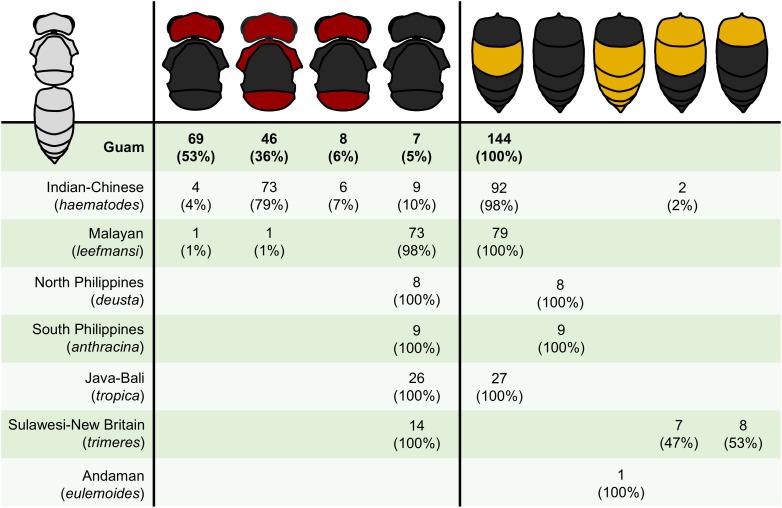
Color profiles of *V. tropica* hornets on Guam did not match the known color forms described across *V. tropica*’s native range. Color profiles for the head+thorax and gaster were estimated for individual hornets across available specimens (images from iNaturalist.org for all native geographic regions; collected specimens and video records additionally for Guam). Within each native region, color forms adhered to Archer’s [33] descriptions. The number of records (and percentage of total) are given for each combination of colors that was observed for either the head+thorax or gaster in a geographic location (row).

**Fig 8:**
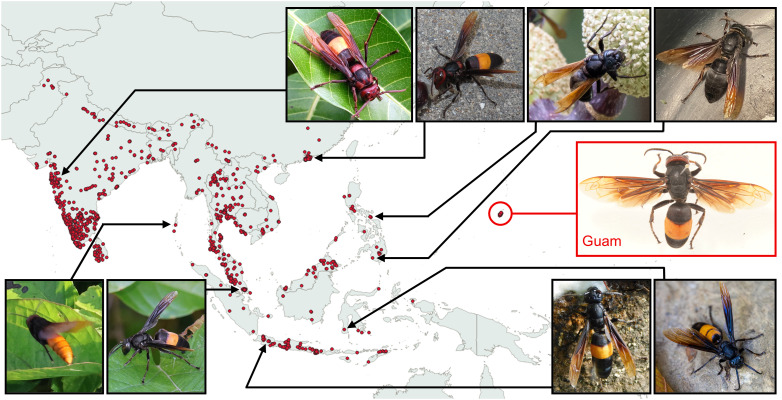
Color forms across *V. tropica*’s native range compared to hornets from Guam. The map shows locations where community scientists have photographed *V. tropica* hornets, affirming its described native range [33] and presence on Guam (based on 2,112 research-grade observations from iNaturalist.org, accessed on July 17, 2025 [46]). Examples of seven *V. tropica* color forms, detailed by Archer [33], are shown (black-bordered images, with arrows to sites), as well as a pinned worker from Guam (red-bordered image, with a circle showing Guam). Top row, left to right: Indian-Chinese forms from India and Hong Kong (*haematodes*); northern Philippines form *(deusta*); southern Philippines and Palawan form (*anthracina*). Bottom row, left to right: Andaman form (*eulemoides*); Malayan form (*leefmansi*); Java and Bali form (*tropica*); Sulawesi-New Britain form (*trimeres*). The Buruan form (*unicolor*), indistinguishable from the southern Philippines form and lacking iNaturalist records, is not depicted. Hornets on Guam were variable, with most color profiles intermediate between *haematodes* and *leefmansi*. Photo credits clockwise from top left: SA Kajawe; YC Tam; F Botial-Jarvis; L Chen; GW Otis and S Paiero; F Botial-Jarvis; G. Cahyadi; GC Weei; J Meyer. The map was generated using QGIS Geographic Information System, whose world map is licensed under the Creative Commons Attribution–Share Alike 3.0 license (CC BY–SA). The authors specify that this figure, with our addition of *V. tropica* location data, is licensed under CC BY 4.0.

Our examination of iNaturalist images throughout *Vespa tropica*’s native range confirmed seven of the eight regional color forms described by Archer [2,33], except *unicolor* of Buru Island, for which no images were available (Fig 7). Exemplars of these seven color forms are depicted in Fig 8. The greatest variability in head, thorax, and gaster color profiles occurred in the continental Indian-Chinese (*haematodes*) and, to a lesser extent, Malayan regions (*leefmansi*), whereas color profiles were relatively unique yet uniform within island-based regions (Fig 7), suggesting that coloration could be a useful tool for detecting the geographic source of invasive *V. tropica* if they originated from any of these islands.

Of the 159 *V. tropica* adults that we examined from Guam, we were able to score dominant colors for most of them (n = 130 for head+thorax colors; n = 144 for gaster colors). Color profiles for hornets’ head+thorax on Guam were highly variable and best matched (but not closely) the mix of color profiles that were found across the Indian-Chinese and Malayan regions (Fig 7). For example, red heads were observed for hornets from these continental regions only. These two continental color forms also aligned with the gaster coloration of invasive Guam hornets, with almost all of them exclusively limited to one color profile (Fig 7). In contrast, Guam hornets did not match any of the distinctive island-based color forms in *V. tropica*’s native range (Fig 7). A lack of clear match with native continental and island color forms means that genetic analyses are necessary to identify the geographic origin of invasive *V. tropica* on Guam. We note that, in several iNaturalist images, body segments of hornets had tones of deep maroon or dark gray that we categorized as black and tones of yellow that we categorized as orange, according to Archer [33]. The full list of specimens, iNaturalist IDs, and categorization details are provided in supporting information (S2 Dataset).

## Discussion

We have provided the first analysis of the impact of invasive *Vespa tropica* hornets on Guam since they were detected there in 2016 [30]. Unlike the failure of many accidentally introduced hornet species to establish invasive populations outside of their native ranges [4], it is clear that *V. tropica* is thriving on Guam. Strong communication between the Guam Beekeepers Association and the biosecurity office (CAR leads both), combined with a culture among island residents of attention to invasive species, have produced a comprehensive set of field reports that describe undiminished *V. tropica* activity on the island over the last nine years. One major takeaway is that, in contrast to temperate *Vespa* species [12,51], *V. tropica* colonies are active on Guam throughout the year, showing no restriction to parts of the year only. This is the first evidence of an asynchronous life cycle for *V. tropica* colonies, as is known for several tropical *Vespa* species [20,42,51]. However, we note that there was still strong seasonality to *V. tropica* activity; observations of nests and hornets, including multiple-hornet attacks on honey bee colonies, were far more likely to be made during Guam’s wet season, which runs from July to December. Because our data rely on public reports, we infer that colonies are more likely to be in a period of growth or be relatively large during the wet season, thus making nest activity or hunting hornets more noticeable as colony food demands and foraging rates increase. We do not have data about seasonality of managed hive losses due to *V. tropica* attack, but it is likely to mirror the increased probability of hornet attacks from July to December. This pattern of activity is important for Guam beekeepers and local officials to know because it will help them to predict when hornets will be more likely to cause damage in apiaries or pose an increased threat to public safety, which can inform beekeeper guidance and public policy.

Another important observation on Guam was the frequent and year-round attacks on honey bee colonies by multiple hornets at the same hive. The first multiple-hornet attack was reported in 2018, when a local beekeeper saw over a dozen hornets raiding his hive on a farm in northern Guam. This site also had the first recorded colony loss due to *V. tropica* attack. Although the degree to which *V. tropica* hornets engage in group hunting remains unclear, *V. tropica*’s repertoire of hunting behaviors is remarkably reminiscent of the well-known predation strategies that are used by giant hornets, *Vespa mandarinia* and *Vespa soror*, as they prey upon social insect colonies [12,13,64,65]. Numerous *V. tropica* workers often gathered at the entrance of a hive, ignoring nearby hives in favor of a single target (Fig 4C). The hornets often killed or injured bees, then dropped them quickly before returning to the hive to keep attacking (Fig 4D). Serial killing by individual *V. tropica* workers is akin to the ‘slaughter phase’ that has been described for both giant hornet species [13,64,65]. *V. tropica* hornets entered and exited weakened or abandoned hives at will, consuming both bee brood and dead workers, which may equate to the final ‘occupation phase’ for which giant hornets are known. Similar behavior by *V. tropica* has been described in two other cases (the only published reports of which we are aware) in which a group of *V. tropica* hornets attacked and then occupied an *A. florea* and an *A. mellifera* colony, both in Thailand [66,67]. On Guam, co-attacking *V. tropica* hornets repeatedly engaged in trophallaxis (S5 Video), as is observed when giant hornet nestmates attack a target colony [13,64]. While it is possible that unrelated hornets are attacking and opportunistically entering the same weakened hive [72], trophallaxis supports the impression that co-attackers were familiar nestmates. When hive entrances were blocked or reduced by beekeepers, *V. tropica* hornets chewed their way through the styrofoam hive boxes that are popular on Guam, similar to the nest-breaching behavior reported for *V. soror* in Vietnam [64]. Another predatory behavior of giant hornets, marking of target prey nests with recruitment pheromones [65,73,74], has been anecdotally reported for *V. tropica* hornets on Guam, but needs confirmation. While we have also observed lone *V. tropica* workers preying upon *Polistes* and *Ropalidia* nests on Guam, as is often observed in its native range [20,42,59], it is striking how frequent group attacks on honey bee colonies were on Guam.

*A. mellifera* colonies on Guam responded to attacks by *V. tropica* workers with defenses they are known to use elsewhere against hornet predators, both in regions of sympatry and where translocations have brought predator and prey into contact. We observed honey bees forming bee ‘carpets’, or clusters of bees on hive surfaces just outside entrances, and, occasionally, trying to bee ball attacking hornets. Tightly packed bees can block nest entrances and give workers a better chance of capturing attacking hornets in bee balls [75,76]. Similar carpeting behavior has been observed from western Europe to the eastern Mediterranean in regions where *A. mellifera* comes into contact with its natural predators, *Vespa crabro* and *Vespa orientalis* [72,75], as well as unfamiliar *V. velutina* [76,77]. We observed only three attempts to ball hornets, none of which were successful. Attempts to ball hornets by *A. mellifera* can be difficult to catch on video without continuous monitoring [78], which we lacked. Reports of dead *V. tropica* hornets inside hives on Guam suggest that bees did successfully ball *V. tropica* workers, although not efficiently enough to prevent colony losses. *A. mellifera* workers are capable of killing hornets in bee balls [72,75,76,78–80], but they do not do it as effectively as *Apis cerana*, which co-evolved alongside many *Vespa* species, including group-hunting hornets [81,82]. On Guam, observations of reduced foraging, or ‘foraging paralysis’, were associated with several colony losses, which is also commonly reported for *A. mellifera* when encountering *Vespa* predators at hive entrances [76,77,79,81,83]. Although we did not observe absconding, the repeated discovery of hives with bee brood but without adults suggests that it happened. While absconding is not a typical behavior for European *A. mellifera* [84,85], it has been observed multiple times when colonies are attacked by giant hornets [86] as well as in response to the only other report of a group attack by *V. tropica* hornets on *A. mellifera* [67]. We saw no evidence that honey bees on Guam made hive entrances smaller using propolis walls, which are created by *A. m. cypria* on Cyprus to defend against sympatric *V. orientalis* predators [72,76].

Coupled with the frequency of multiple-hornet attacks, another surprising finding was the strong piping response of *A. mellifera* workers when their colonies were being attacked by either single or multiple *V. tropica* workers. Presently, our available audio is limited to relatively low-quality cell phone recordings, but even in these videos bees can be heard making repeated and overlapping pipes (S6 Video). Individual pipes were variable in duration and, while some of them were brief like typical stop signals, many of them were longer. Stop signals, which *Apis* workers may use when they perceive predator danger outside hives [87], including *A. cerana* in response to hornets [77,88,89], are usually short (mean 142–258 ms across studies) and often, but not always, delivered via head butts to waggle dancing recipients [87,88,90–93]. Most of the vibroacoustic signals we analyzed were relatively longer (Fig 6B) and the pipers we identified in videos all delivered signals by pressing their thorax onto landing boards or other workers in the bee carpets outside hive entrances. Many of the pipes had acoustic features of antipredator pipes (longer duration, strongly modulated frequency, and harmonic structure mixed with broadband energy), sharing properties of the alarm signals used by *A. cerana* in response to attacks by *V. soror* giant hornets [89] or by *A. m. cypria* before bee-balling *V. orientalis* attackers [94]. On Guam, we observed a body posture similar to that reported for introduced *A. mellifera ligustica* workers in Japan when piping in response to native *Vespa simillima* hornets (see [95]), rather than the racing, wing-buzzing, abdomen-aloft posture of alarmed *A. cerana* workers confronted with *V. soror* attackers [89]. Notably, the *A. mellifera* pipers we saw outside hive entrances did not move with urgency, despite the predatory threat they faced, even when they walked to the front line of the bee carpet and were in close proximity to *V. tropica* hornets. Overall, their movements lacked the visible alarm that is conveyed when *A. cerana* workers pipe in response to giant hornet attacks. Further study is needed to determine how the actions of signaling *A. mellifera* workers affect recipients as colonies confront hornets on Guam.

Our observations of predator-prey interactions on Guam revealed more robust attack and defense behaviors than might be expected for two introduced species encountering one another on a remote island, although some elements of their interaction revealed their predator-prey mismatch. Because *A. mellifera* evolved under substantially lower predation pressure from hornets than Asian honey bee species, they tend to respond relatively poorly when they are brought into contact with new *Vespa* predators [76,77,86,96–99], which is reflected in hornet-bee interactions on Guam. Guam’s honey bees are derived from western Europe, having arrived in 1907 from stock that was imported to Hawaii several times during the 1800s from western Europe [100,101]. Further import of *Apis* to Guam was prohibited after 1956 [102]. Guam bees are genetically dominated by haplotypes that characterize *Apis mellifera carnica* (Carniolan stock) [103], making it likely that the island’s honey bees were ancestrally exposed to *V. crabro* and possibly *V. orientalis* as native hornet predators. It is fascinating that *A. mellifera* colonies on Guam show a colony-level response to hornet attack, although it appears limited to a level of efficacy that may fend off solitary-hunting *Vespa* species only [75]. Equally fascinating, while piping by *A. mellifera* in response to *V. crabro* has not been reported, colonies derived from European stock are capable of piping in response to hornet attack (i.e., bees on Guam, introduced *A. m. ligustica* in Japan and China: [77,95]; native *A. m. cypria* on Cyprus [94]). On the other side of this interaction, it is intriguing that *V. tropica* attacks honey bees in apparently cooperative groups on Guam, yet they are not widely reported to hunt this way in their native range [20,33,42,63]. Furthermore, they do not seem to be especially proficient group hunters. On Guam, as in other limited reports of group attacks by *V. tropica* [66,67], co-hunters attacked colonies over many days, seemingly winning occupation after slow attrition through constant harassment. This is in contrast to the highly efficient group attacks of giant hornets, which inevitably overcome *A. mellifera* colonies, usually within hours after initiating an attack, wherever *A. mellifera* is introduced into the former’s range and whenever they are left unprotected by beekeepers [86,104]. Overall, it means *V. tropica* predation on Guam is a problem that beekeepers need to manage, but they have a chance to save molested colonies with apiary vigilance.

Questions inevitably arise about the geographic source of invaders as part of understanding the status and impact of any invasion [105]. Currently, a genetic database of sequenced *V. tropica* samples does not exist, making it difficult to estimate propagule pressure or identify the native population that gave rise to the invasion on Guam [4,106], as has been explored for other *Vespa* invasions [25,29,107–112]. However, there is an ongoing effort to collect and genetically analyze specimens from *V. tropica*’s vast native range to compare with specimens from Guam. In the meantime, we wondered whether color forms could be informative, given that several of them are distinctive or specific to islands, which may help to eliminate the color gradations that make melanisation difficult to link to geographic origin in other *Vespa* species [113]. We found that Archer’s [33] geographical descriptors of *V. tropica*’s eight color forms were confirmed by community scientists’ images from across its geographically broad range [46], suggesting that color may be a potentially useful trait for identifying the source of invasive *V. tropica* in certain scenarios (e.g., if Guam specimens had one of the unique color forms linked to islands in *V. tropica*’s native range). However, specimens from Guam were variable and collectively did not align well with any native color form, showing that genetic analyses are essential for identifying invader origin. While exercising caution about overinterpreting these results, it is unlikely that Guam’s invaders originated from the islands of the Philippines, the Andaman Islands, or from most parts of Indonesia, regions where coloration is strikingly different from hornets observed on Guam. Guam hornets had a mix of color profiles that aligned most closely (but not exactly) with continental Indian-Chinese and Malayan color forms (*haematodes* and *leefmansi*), the two groups that Archer [33] described has having intermediate color combinations. These color forms cover a substantial geographic range, prohibiting us from pinpointing the potential origin of *V. tropica* hornets on Guam. Many other questions about propagule pressure remain to be answered by anticipated genetic analyses, including the possibility that *V. tropica* was introduced more than once or as a colony that had more than one queen [42,49,109,114]. We also cannot exclude the possibility that *V. tropica* has a high tolerance for other hornets at hive entrances on Guam because low levels of genetic diversity within the introduced population have resulted in reduced inter-colony aggression due to similarity in nestmate recognition cues. While the inbred, invasive population of *V. velutina* in Europe retains surprisingly heterogenous cuticular hydrocarbon profiles among colonies despite a single introduction event [115], streamlined chemical profiles are thought to play a role in the success around the world of several species of highly invasive ants [116–120].

What can the residents of Guam expect from this established invasion by *V. tropica*? One major impact is the predation pressure that *V. tropica* likely places on the island’s entomofauna. As a known semi-specialist of social wasps and honey bees [20,36,42,45,59,63], its predominant food resources on Guam may be limited. We have observed *V. tropica* preying on social wasps on Guam (S10 Video). It is our impression, based on our own experience (CAR and RHM) and anecdotal reports from Guam residents, that the formerly ubiquitous ‘boonie bees’, the colloquial term for Guam’s non-native eusocial wasps (*Polistes stigma*, *Polistes olivaceus*, and *Ropalidia marginata*), are now uncommonly encountered, although data confirming this decline or its cause are lacking. We predict that *V. tropica* opportunistically exploits many types of prey on Guam, given the adaptable and cosmopolitan prey palate of invasive *V. mandarinia* and *V. velutina* in new habitats [14–19]. Based on our findings, we also predict this predation pressure would continue all year, but be strongest during the July-December wet season, when nest visibility and multiple-hornet attacks are heightened on Guam. Further, we predict that *V. tropica* activity would decrease if particularly wet weather damages nests, as has happened in *V. tropica*’s native range [68]. The strongest storm in decades, category 4 Typhoon Mawar, hit Guam in 2023, which may explain the dip in *V. tropica* reports that occurred that year (Figs 2A and 5). We also note that, although surveys of the size of *V. tropica* nests are limited to three nests in its native range [38,42] and three nests from Guam, nests in its native range were larger (2,580–5,667 cells versus 650–1,350 cells, respectively), which may be related to prey availability. More data are necessary to confirm the size of mature *V. tropica* nests on Guam, which have a natural potential to be quite large. Importantly, Guam residents should expect to encounter nests and hornets everywhere, including residential properties and greenspaces, both below and above ground, in cavities and in the open. Our finding that *V. tropica* are more commonly found in association with urban and residential landscapes on Guam is likely an artifact of public reporting. Our guess is that *V. tropica* commonly nests in Guam’s lowland farmland and forests, as they do in their native range [20,35,36,42,47], but they are less frequently encountered because of the impenetrability of Guam’s hilly forests.

Beekeepers, researchers, and biosecurity officials on Guam have explored several strategies for managing the impacts of invasive *V. tropica* hornets. The first line of defense has been destroying nests that are reported by the public, although our study makes clear that the invasive population has not been eradicated by this effort. In apiaries, some beekeepers screen hive entrances, which physically separates predator and prey. However, screens can make it hard for bees to forage freely and the presence of hornets at entrances may induce foraging paralysis, which can decrease colony productivity under even low levels of predation threat by other *Vespa* species [77,78,83,121]. To manage predatory pressure and its consequences, beekeepers often resort to killing hornets by hand, which is viewed as an unsustainable tactic. For instance, one beekeeper who kept a careful count killed over 1,000 hornets during a 10-week period in a single apiary with four hives. Many beekeepers simply move molested hives to escape predation pressure. Researchers and beekeepers have field tested one-way entrance traps that allow hornets to enter but not leave, similar to the hornet traps tested in Europe and Asia [86,104,122]. To date, a highly effective entrance-trap design has not been identified. Hornet baits, such as a modified version of the McPhail fruit fly trap, have also been tested to see whether they can mitigate hornet predation in apiaries and monitor hornet presence at the Guam port-of-entry. Unfortunately, these efforts have not yielded a bait trap that avoids unselective insect bycatch, a problem observed in similar hornet bait traps that have been tested in Europe [123]. Beekeepers, researchers, and government officials continue to collaboratively test potential traps that effectively capture hornets at hive entrances and avoid killing nontarget insects.

**Fig 9:**
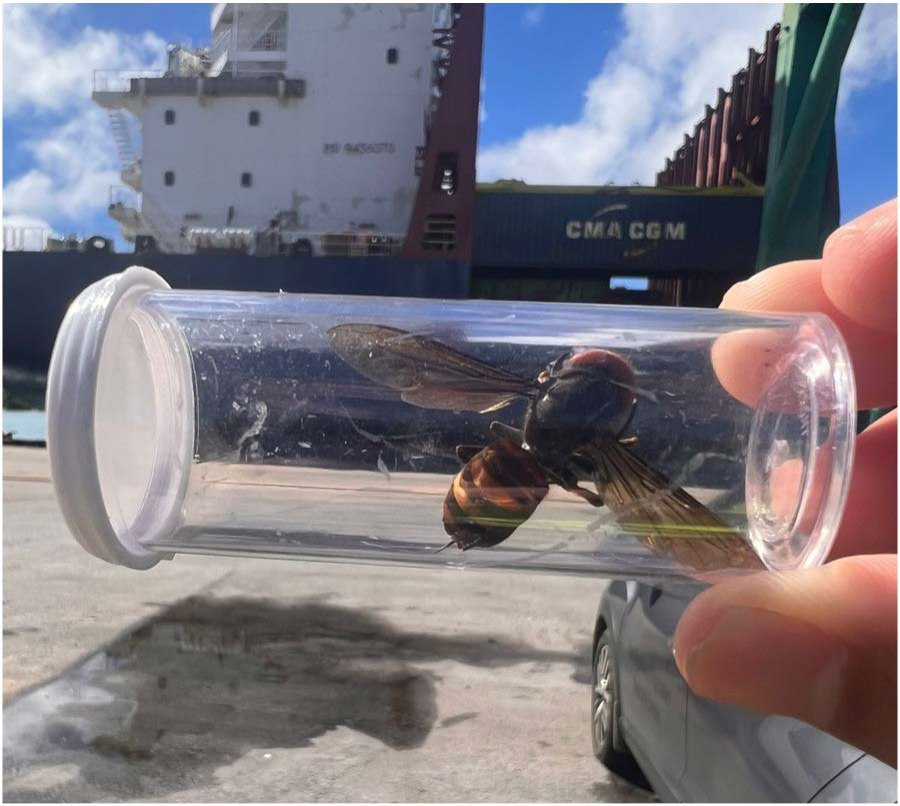
A *Vespa mandarinia* adult was recently intercepted by the Port Authority of Guam. A dead female giant hornet was discovered by an Environmental Compliance Specialist in a cargo container on October 18, 2023. A live inseminated gyne could found an entire colony.

Guam has long experienced accidental introductions of exotic wildlife [124], often with devasting results. The most widely known invasive, the brown tree snake (*Boiga irregularis*), has destroyed the island’s avifauna and is the cause of frequent power outages [125,126]. Over a 15-year period, harmful insects were accidentally introduced to Guam at a rate of 1.5 detected species per year, with the true rate, including undetected species, estimated at 10–12 species per year [127]. These invaders originated from Asia, Hawaii, other islands of Micronesia, and mainland United States; Guam is also a source for many species that get accidentally introduced to other islands within Micronesia [128]. Unfortunately, based on a notorious history to date with the accidental introduction of damaging invasives [129], Guam residents should expect continued propagule pressure from non-native species, including other species of *Vespa*. For instance, a *V. mandarinia* adult was recently intercepted by the Port Authority of Guam during a routine inspection in October 2023 (Fig 9, [130]). In 2016, the year that *V. tropica* was first detected on Guam [30], Gaum’s Port Authority received over 100,000 cargo containers [131]. As a major transportation hub for the western Pacific region and with trade routes originating from jurisdictions where hornets are common—for example, China (including Hong Kong), South Korea, Japan, the Philippines, Vietnam, Thailand, Malaysia, and Singapore [132]—Guam is positioned to receive more *Vespa* invaders.

## Supporting information

S1 Supporting Information Details about photos in figures, including photographer and location. (DOCX)

S2 Dataset All raw data for this study, including anonymized reports. (XLSX)

S3 Video *V. tropica* hornet grabbing an *A. mellifera* worker from the edge of a bee ‘carpet’. Filmed in slow motion. (MP4)

S4 Video Two *V. tropica* hornets killing *A. mellifera* workers during a multiple-hornet attack. (MP4)

S5 Video Multiple instances of trophallaxis between *V. tropica* hornets during attacks on *A. mellifera* colonies. (MP4)

S6 Video *A. mellifera* workers piping during a *V. tropica* attack. Worker piping is audible if the volume is maximized. (MP4)

S7 Video An *A. mellifera* worker pipes another worker in a bee carpet. A red arrow indicates a piping worker before she pipes; the word “piping” is visible when she pipes. (MP4)

S8 Video An *A. mellifera* worker piping as she moves around a bee carpet and approaches attacking *V. tropica* hornets. Red arrows indicate each time the worker stops walking to pipe. (MP4)

S9 Video *A. mellifera* bees make two attempts to engulf *V. tropica* hornets in bee balls. (MP4)

S10 Video A lone *V. tropica* worker eating a *Polistes stigma* pupa on a nest on Guam. Adult *P. stigma* workers move off of their nest during the attack. (MP4)
