# Supplementary material for: Predatory behavior, nesting habits, and impacts on honey bees (Apis mellifera) of an invasive hornet (Vespa tropica) on the island of Guam
Source: PLoS One. 2025 Sep 26;20(9):e0332986. doi: 10.1371/journal.pone.0332986 (PMC12469110; doi:10.1371/journal.pone.0332986)
Supplement: S1 Supporting Information — (DOCX) [file pone.0332986.s001.docx]

*Vespa tropica* photo sources (permissions) and locations:

**Guam specimen:**

**Gard W. Otis / Steve Paiero**

Specimen collected by Chris Rosario in Barrigada, Guam (13.465 N, 144.798 E; June 1, 2024)

***V. tropica haematodes*, west end of distribution:**

**Sanjana Arvind Kajawe,**

-iNaturalist.org user name = “sanjana11”); observation #102697840

-India: Mahajarashtra, near Nashik (19.95N, 73.77E; December 8, 2021).

***V. tropica haematodes*, east end of distribution**:

**Yu Ching Tam**

-iNaturalist.org username = “yuchingtam”); observation #178518610

-China: Hong Kong, Lantau Island (22.25N, 113.91 E; August 8, 2023)

***V. tropica deusta***, **northern Philippines:**

**Forest Botial-Jarvis**

-iNaturalist.org user name = “tiluchi”; observation #145236158

-Philippines: Sorgoson Province, ~13 km WNW of Sorgoson City (13.021N, 123.916E; April 25, 2024)

***V. tropica anthracina***, **southern Philippines:**

**Larry Chen**

-iNaturalist.org user name = “cypselurus”); observation #143684777

-Philippines: Mindanao, Sultan Kudarat Province, Senator Nonoy Aquino County, Kulaman (6.47N, 124.33E; August 27, 2022)

***V. tropica trimeres***, **Sulawesi/New Britain (includes form *trisigmata*) of Sulawesi, Flores, Sumba, Timor, to New Britain:**

**Forest Botial-Jarvis**

-iNaturalist.org user name = “tiluchi”; observation #191529868

-Indonesia: South Sulawesi, Maros Regency, Bantimurung/Bulusaraung National Park (5.03S, 119.75 E; September 25, 2023)

***V. tropica tropica*, Java-Bali form:**

**Ganjar Cahyadi**

-iNaturalist.org user name = “ganjarcahyadi”; observation #140960963

-Indonesia: Jawa Barat Prov., Sumedang Regency, Cileunyi, 15 km east of Bandung

**(**-6.93 S; 107.77 E; November 3, 2022)

***V. tropica leefmansi*, Malayan form:**

**Gan Cheong Weei**

-iNaturalist.org user name = “gancw1”; observation #104574543

-Singapore: Windsor Park (1.356N, 103.823E; January 5, 2022)

***V. tropica eulemoides*, Andaman Island form:**

**Jonathan Meyer**

-iNaturalist.org user name = “isidoreajar”; observation #198330625

-India: Andaman and Nicobar Islands, South Andaman Islands, Bamboo Flat (11.72N, 92.73E; January 17, 2024)

***V. tropica unicolor***: Buru form; indistinguishable from *anthracina* (Archer, 1991).

(no photo available)

***V. tropica* with *V. soror*, size comparison**

**Jonathan Yang**

-iNaturalist.org user name = “robotpie”; observation #14129950

-China: Hong Kong, Tuen Mun District, Lung Kwu Tan (22.39N, 113.92E; July 6, 2018)

***V. tropica* attacking colony of *Ropalidia* sp.**

**Vikrant Kumar**

-iNaturalist.org user name = “vikrantk”); observation #128256645

-India: Karnataka State, Bangalore District, Bengaluru (12.96N, 77.60E; July 28, 2022)
